# mHealth Apps for Musculoskeletal Rehabilitation: Systematic Search in App Stores and Content Analysis

**DOI:** 10.2196/34355

**Published:** 2022-08-01

**Authors:** Shíofra Ryan, Noirín Ní Chasaide, Shane O' Hanrahan, Darragh Corcoran, Brian Caulfield, Rob Argent

**Affiliations:** 1 School of Public Health, Physiotherapy and Sports Science University College Dublin Dublin Ireland; 2 Insight Centre for Data Analytics University College Dublin Dublin Ireland; 3 School of Pharmacy and Biomolecular Sciences Royal College of Surgeons in Ireland University of Medicine and Health Sciences Dublin Ireland

**Keywords:** mHealth, musculoskeletal rehabilitation, app, home exercise program, home exercise, telehealth, mobile health, connected health

## Abstract

**Background:**

The number of mobile health (mHealth) apps released for musculoskeletal (MSK) injury treatment and self-management with home exercise programs (HEPs) has risen rapidly in recent years as digital health interventions are explored and researched in more detail. As this number grows, it is becoming increasingly difficult for users to navigate the market and select the most appropriate app for their use case. It is also unclear what features the developers of these apps are harnessing to support patient self-management and how they fit into clinical care pathways.

**Objective:**

The objective of this study was to scope the current market of mHealth apps for MSK rehabilitation and to report on their features, claims, evidence base, and functionalities.

**Methods:**

A cross-sectional study of apps for MSK rehabilitation was performed across the iTunes App Store and Google Play Store. Four search terms were used, namely, physiotherapy rehabilitation, physical therapy rehabilitation, rehabilitation exercise, and therapeutic exercise to identify apps, which were then cross-referenced against set selection criteria by 4 reviewers. Each reviewer, where possible, downloaded the app and accessed supplementary literature available on the product to assist in data extraction.

**Results:**

A total of 1322 apps were identified. After applying the inclusion and exclusion criteria and removing duplicates, 144 apps were included in the study. Over half (n=81, 56.3%) of the included apps had been released within the past 3 years. Three quarters (n=107, 74.3%) of the apps made no reference to evidence supporting the design or efficacy of the app, with only 11.1% (n=16) providing direct citations to research. Most of the apps did utilize exercise pictures (n=138, 95.8%) or videos (n=97, 67.4%); however, comparatively few harnessed additional features to encourage engagement and support self-management, such as an adherence log (n=66, 45.8%), communication portal (n=32, 22.2%), patient-reported outcome capture (n=36, 25%), or direct feedback (n=57, 39.6%). Of note and concern, many of these apps prescribed generic exercises (n=93, 64.6%) in the absence of individualized input to the user, with few providing specific patient education (n=43, 34%) and safety advice or disclaimers (n=38, 26.4%).

**Conclusions:**

The cohort of apps included in this study contained a large heterogeneity of features, so it is difficult for users to identify the most appropriate or effective app. Many apps are missing the opportunity to offer key features that could promote exercise adherence and encourage self-management in MSK rehabilitation. Furthermore, very few developers currently offering products on the market are providing evidence to support the design and efficacy of their technologies.

## Introduction 

An injury to the musculoskeletal (MSK) system involves damage to 1 or more components of the locomotor system and its associated tissues. These injuries account for the greatest proportion of noncancer persistent pain conditions [1]. The World Health Organization (WHO) estimates that between 20% and 33% of people across the world live with a painful MSK condition, and it is the highest contributor to global disability, with low back pain the single leading cause of disability worldwide [1,2] The burden of MSK conditions on societal and personal well-being is escalating, resulting in a reduction of quality of life, mental well-being, and function [3]. Additionally, MSK conditions account for 25% of overall costs of illness globally, placing a significant burden on health care resources [4]. Exercise as treatment for MSK conditions is widely accepted [5], with clinical guidelines advocating the promotion of physical activity and the use of exercise programs [6,7]. 

The prescription of home exercise programs (HEPs) encourages patients to take responsibility and self-manage their condition to mitigate limitations in physical function, a hallmark consequence of MSK conditions [8]. Adherence is considered an important prerequisite for the success of HEPs and has a direct link to improved patient outcomes [9]. However, in a study by Bassett et al [10], nonadherence to HEPs was estimated to be as high as 50%. Therefore, solutions to improve adherence and support self-management are required to optimize the efficacy of MSK treatment [11,12]. It has been suggested that mobile apps and connected health technologies can incorporate design features to maximize adherence, encourage self-management, and bridge the gap between the clinic and home [11,13]. 

Mobile health (mHealth) is defined by the WHO Global Observatory for eHealth as a “medical and public health practice supported by mobile devices, such as mobile phones, patient monitoring devices, personal digital assistants, and other wireless devices” [14]. The current capabilities and ubiquity of mobile devices make them a valuable tool for improving the delivery of health care services and providing scalable, low-cost interventions [15]. Today, 3.8 billion people worldwide own a smartphone [16], posing an opportunity for health care providers to make health care accessible to a large proportion of the population [17,18]. With this rise in accessibility of mHealth comes a surge in the choices of apps, with at least 318,000 health apps available worldwide [19]. Other areas of health, including diabetes and hypertension management, have reported promising results in favor of the use of apps for improving several clinical, behavioral, knowledge, and psychosocial outcomes [20,21]. With the mHealth app market growing exponentially, the employment of such apps in various clinical contexts correlates with this growth. Clinicians in both cardiac and neurorehabilitation/palliative care adopting mHealth apps into their practices have reported similar, clinically relevant successful outcomes [22,23].

One clear use case that mHealth affords health care professionals is the opportunity to provide interactive and engaging access to self-management programs for MSK rehabilitation, incorporating features such as goal setting, coaching, remote monitoring, and exercise tracking [11,24]. Therefore, these systems have the potential to increase self-efficacy, optimize quality of life, and reduce the burden of MSK conditions [25,26]. However, caution must be taken with this opportunity, as there is a need for better standardization and regulation of mHealth apps to ensure proper integration and identification of beneficial and safe apps [27,28]. With over 200 health apps being added to the iOS and Google Play app stores each day [17], the integrity, in terms of quality and safety, of mHealth apps is questionable. Despite the iTunes App Store and Google Play Store categorizing apps (health, well-being), searches on the stores yield millions of results of indeterminate quality [29,30], making the search and selection of health care apps challenging for clinicians and patients alike [31]. There is a large body of qualitative research looking at the potential of mHealth to improve adherence and the role that digital technology can play in exercise rehabilitation [32,33]. However, research examining the current state of mHealth apps for exercise rehabilitation is limited. A recent systematic review found that approximately one-third of the 102 studies included evaluated the clinical efficacy of an intervention, with the remainder assessing the functionality of the app or patient engagement with the app [34]. To our knowledge, there has been no research to date exploring the overall scope of the market.

Given the exponential rise in mHealth apps and the limited research into their effectiveness and acceptance, the aim of this study was to investigate the current state of the mHealth app market targeted at assisting patients with MSK exercise rehabilitation. Recent innovations in health care provision can help improve the delivery and efficacy of physiotherapy to this cohort of patients [35]. The aim of this paper is to scope the current market of mHealth apps for MSK rehabilitation and describe which features exercise rehabilitation apps currently offer, document the accessibility of the app, and explore the evidence supporting each individual app. 

## Methods

### Study Design

A cross-sectional study of MSK rehabilitation apps was performed to identify apps from 2 major smartphone app stores: iTunes App Store and Google Play Store, which together represent 98.9% of the smartphone app market share [36]. Building on the approach by Giunti et al [27], a systematic search strategy was developed that attempted to identify all relevant apps, followed by a synthesis of the characteristics of the apps. 

### Setting

On October 28, 2020, 4 reviewers searched both stores from the Republic of Ireland using 4 different search terms: “physiotherapy rehabilitation,” “physical therapy rehabilitation,” “rehabilitation exercise,” and “therapeutic exercise.” The iTunes App Store is a digital distribution platform developed and maintained by Apple Inc for mobile apps on iOS with 1.96 million apps available [16]. Google Play store (originally the Android Market) serves as the official app store for the Android operating system and contains over 2.86 million apps [16]. 

### Selection Criteria

Apps were included if they were available in English, focused on exercise interventions for MSK injuries or general MSK physiotherapy rehabilitation, and were available for use on smartphone devices. Apps that were determined to be general well-being/fitness apps without reference to physiotherapy or rehabilitation were excluded. A full list of inclusion and exclusion criteria can be found in Textbox 1. 

**Inclusion and exclusion criteria.**

**Inclusion criteria**

- Title or description makes reference to musculoskeletal (MSK) physiotherapy/physical therapy rehabilitation
- Title or description makes reference to exercise interventions for specific or general MSK conditions
- Patient-centered app
- Includes exercise prescription

**Exclusion criteria**

- Title or description does not make reference to MSK physiotherapy/physical therapy rehabilitation
- Title or description does not make reference to exercise interventions for specific or general MSK conditions
- Description is not written in English
- Duplicates from the same store
- Clinician-focused app
- Women’s health apps such as pelvic floor center apps
- General fitness apps with no mention of physiotherapy/physical therapy or MSK conditions

After the search was completed, the resultant apps were screened by 1 of 4 reviewers (authors SR, NNC, SOH, and DC) for eligibility against the inclusion and exclusion criteria. A small random sample (5%) was independently reviewed by 2 reviewers who evaluated the eligibility of the apps against the selection criteria. To assess the clarity of the selection criteria, interrater reliability was assessed using Cohen kappa coefficient. If any conflicts or disagreements arose, the app in question was discussed between the 4 reviewers until they came to an agreement. In line with common practice, different versions of the same app (basic/premium, iOS/Android) were included separately due to version capabilities or store submission processes [37]. A cohort of identical apps from the same developer was classed as “white labeled” by the authors, with the underlying app being identical but branded for different health care providers. During selection, this cohort was represented by 1 randomly selected app per developer from each store. 

### Data Extraction

All apps included for data extraction were split evenly between the 4 reviewers. The data were extracted from the app description on the stores, screenshots on the stores, and the app website (link provided on app stores). Data extracted on the apps included year of release, developer, charging models, targeted body part, features, and evidence. If information on the app features was unavailable or unclear in the description, screenshots, and website, the app was downloaded to decipher the remaining features. If a reviewer was unsure of any data, a discussion was held between the 4 reviewers until a resolution was reached. A list of parameters that were used for data extraction and a description of each is included in Multimedia Appendix 1.

## Results

### App Selection

**Figure 1:**
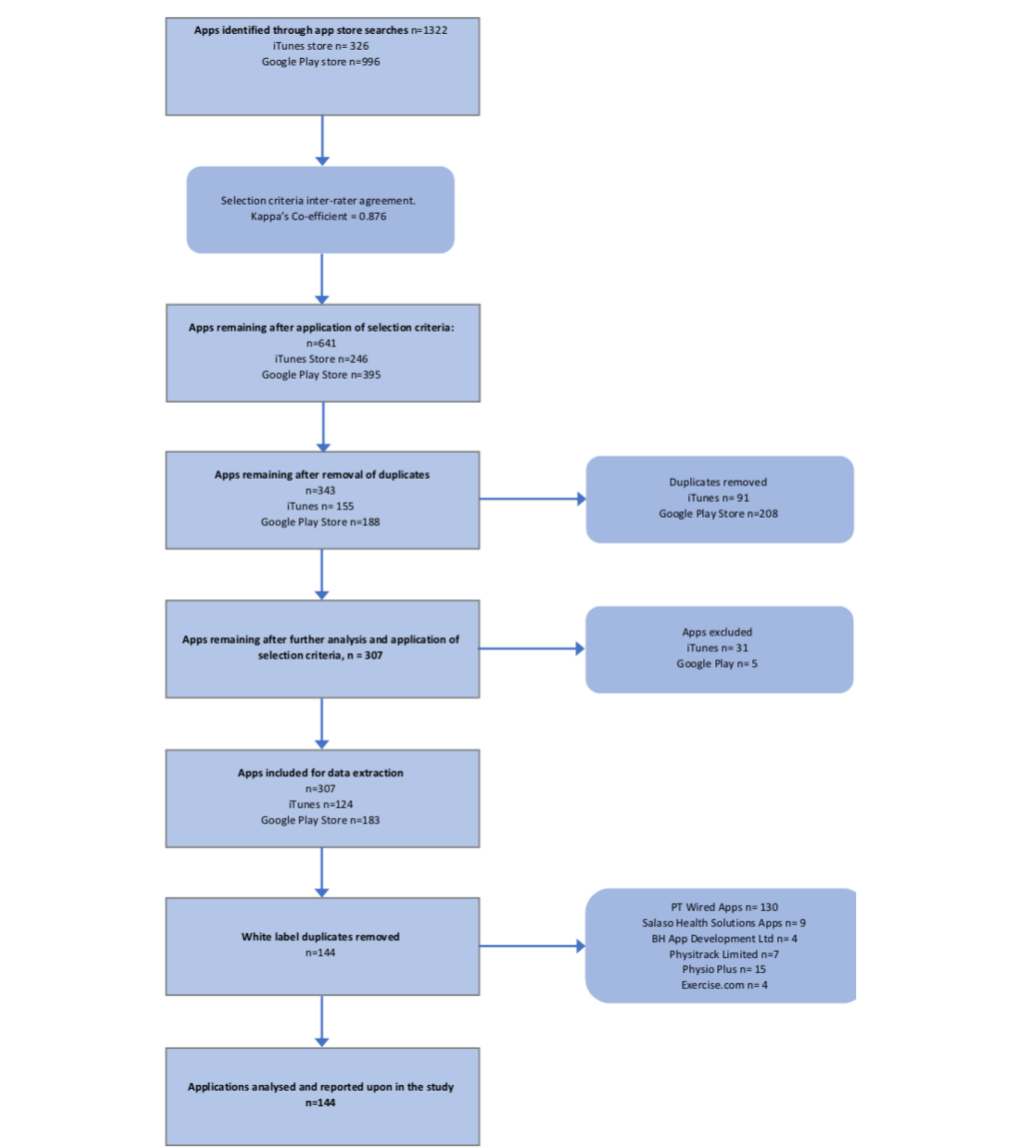
Study Flow.

A total of 1322 apps (326 iTunes App Store and 996 Google Play Store) were identified using the described search strategy. After screening, a total of 641 apps (246 from iTunes App Store and 395 from Google Play Store) met the inclusion/exclusion criteria. Duplicates were then removed, bringing the total to 343 apps. During data extraction from the apps and their associated websites, a further 36 apps were excluded because further investigation revealed that they did not meet the selection criteria. White-label app duplicates were then removed, resulting in a total of 144 apps for data analysis (40 from iTunes App Store and 104 from Google Play Store). Interrater reliability on data screening was tested by calculating the kappa coefficient, resulting in a value of 0.876. Figure 1 presents the flowchart of the app selection process.

### App Analysis Results

#### General Characteristics of Included Apps

The content analysis of the included apps is shown in Table 1. Despite the existence of both app stores since 2008, the past 5 years account for 84% of all app releases, with a notable increase between 2017 and 2019.

The predominant revenue model was a fully cost-free business-to-consumer approach (n=53, 36.8%), with revenue presumably derived from advertising and marketing. A further 23.6%

(n=34) of apps were free to download but offered in-app purchases. This included paying for additional exercises, exercise progression, or other features such as exercise logging. Many other developers (n=43, 29.9%) have pursued a business-to-business model whereby clinics pay for the platform and then provide it in their service to patients. 

**Table 1:** Results of review of apps for musculoskeletal rehabilitation (N=144).

Characteristics		Value, n (%)
**Year of app release**		
	2006	3 (2.1)
	2008	3 (2.1)
	2012	2 (1.4)
	2013	2 (1.4)
	2014	13 (9.0)
	2015	8 (5.6)
	2016	16 (11.1)
	2017	16 (11.1)
	2018	23 (16.0)
	2019	32 (22.2)
	2020	26 (18.1)
**Charging model type**		
	Free to download and no in-app purchase	52 (36.8)
	Clinic charged	42 (29.9)
	Free to download with in-app purchase	33 (23.6)
	Download charge for patient	10 (6.9)
	Multiple	3 (2.1)
	Unable to determine	4 (2.7)
**Evidence base**		
	No research highlighted	107 (74.3)
	References provided to relevant research	16 (11.1)
	Evidence based claims but no reference	21 (14.6)
**Method of exercise prescription**		
	Generic	93 (64.6)
	Tailored to user requirements	44 (30.6)
	Both	7 (4.7)
**Targeted body part**		
	Shoulder	6 (4.2)
	Neck and shoulder	2 (1.4)
	Neck	5 (3.5)
	Knee and hip	2 (1.4)
	Knee and back	1 (0.1)
	Knee	21 (14.6)
	Hand and wrist	1 (0.1)
	Hand	3 (2.1)
	Back and neck	2 (1.4)
	Back and knee	1 (0.1)
	Back and hip	1 (0.1)
	Back and core	1 (0.1)
	Back	18 (12.5)
	Ankle	2 (1.4)
	Tailored	73 (50.7)
**Presence of design enhancing features**		
	Pictures	138 (95.8)
	Videos	97 (67.4)
	Self-reported log	66 (45.8)
	Adherence reminders	49 (34.0)
**Patient-reported outcomes**		
	Standardized instruments	21 (14.6)
	Response to targeted questions	2 (1.4)
	Present—unable to determine method	1 (0.1)
	Free text	1 (0.1)
	Multiple	11 (7.6)
	None	108 (75.0)
**Communication features**		
	Video conferencing	6 (4.2)
	Two-way messaging	8 (5.6)
	Robotic messaging	1 (0.1)
	Messaging—unable to differentiate	2 (1.4)
	Instant messaging	4 (2. 8)
	Multiple sources	11 (7.6)
	None	112 (77.8)
**Feedback to patients**		
	Automated	5 (3.5)
	Progress tracking	25 (17.4)
	Gamification	5 (3.5)
	Direct feedback from physiotherapist	5 (3.5)
	Multiple	17 (11.8)
	None	87 (60.4)
**Clinical specificity**		
	Clinic-specific	18 (19.4)
	Public access	116 (80.6)

Almost three quarters (n=107, 74.3%) of apps made no reference to research or an evidence base for their interventions, nor did they make scientific claims about their apps. Meanwhile, 11.1% (n=16) of the apps did provide research evidence to support the clinical relevance of their platform, marketing claims, or features of the app. The remaining 14.6% (n = 21) of apps made evidence-based claims but failed to reference or supply links to the relevant research.

The majority (n=116, 80.6%) of apps were available to the general population, with the remainder being restricted to a specific clinic and requiring patients to log in to access the features available. Table S1 in Multimedia Appendix 2 shows the general characteristics of the apps.

#### Prevalence of Exercise Prescription and Assistance Features 

Most (n=93, 64.6%) of the apps used automated exercise prescription to generate a generic HEP, while only 30.6% (n=44) of the apps prescribed exercises selected by a health care professional to the patient post assessment (Table 1). The remaining 4.7% (n=7) utilized both methods of exercise prescription. Just under half (71/144, 49.3%) of the apps only targeted the rehabilitation of a specific body part. The knee (n=21, 14.6%) was the most commonly featured body part, followed by the back (n=18, 12.5%) and shoulder (n=6, 4.2%). The remainder (n=73, 50.7%) of the apps did not target a specific body part or tailor the HEP to the specific needs of the individual patient.

Over two-thirds (n=97, 67.4%) of the apps included videos to illustrate the exercise and assist with technique, while the vast majority (n=138, 95.8%) incorporated static pictures in their HEPs. Less than half (n=66, 45.8%) of the apps utilized a self-reported exercise log, although adherence reminders were more frequently used, featuring in 66.7% (n=96) of the apps (Table 1). Table S1 in Multimedia Appendix 2 shows the exercise prescriptions and assistance features of the apps.

#### Prevalence of Communication and Feedback Features 

 Only 25% (n=36) of the apps offered patient-reported outcome (PRO) features supporting self or remote monitoring. Even fewer (n=32, 22.2%) had any direct personalized communication feature. Less than half (39.6%, n=57) included a feature for feedback from the app to the patient. Only 34% (n=49) contained patient education on their app, with fewer (n=38, 26.4%) featuring any safety advice or warnings. 

In the apps containing PROs, standardized instruments like a visual analogue scale or a Likert scale (n=21, 14.6%) were the most common. Only 1.4% (n=2) included specific questions for the patient to respond to, and 0.7% (n=1) included free-text boxes for the patients. Meanwhile, 7.6% (n=11) used more than 1 of these features. It was not possible to determine whether PRO features were used in 0.7% (n=1) of the included apps.

The most common (5.6%, n=8) communication feature was 2-way text messaging between health care professionals and patients (Table 1), followed by video conferencing (n=6, 4.2%), instant messaging (n=4, 2.8%), and robotic automated messages (n=1 0.7%). More than 1 type of communication feature was seen in 7.6% (n=11) of the apps. In 1.4% (n=2) of the apps, it was not possible to identify which communication features were present or if there were any at all.

Of the apps that did include a feature to enable feedback to the patient, progress tracking was the most prevalent (n=25, 17.4%). This is where the patient could track the exercises or workouts they had completed on a calendar. Gamification was utilized in 3.5% (n=5) of the apps, where awards or badges were given. The same percentage of apps supplied direct feedback on progress from the health care provider and included automated feedback, meaning they would receive feedback on their progress through automated messages or emails. Overall, 11.8% (n=17) of the apps included 1 or more of the above feedback-supporting features. Table S2 in Multimedia Appendix 2 shows the additional features of the apps.

## Discussion

### Principal Findings

The sheer volume of mHealth apps available for exercise rehabilitation proves the popularity and prospects of technology in physical medicine. Yet, the acceptance of mHealth apps into routine clinical practice lags behind [38], as clinicians struggle to identify and select appropriate evidence-based apps. This study is the first to complete an in-depth analysis of exercise rehabilitation apps to help elucidate the state of the mHealth app market and investigate the relevance, design, and accessibility of the apps currently available in the iTunes and Google app stores. Despite the prevalence of these apps, many fail to offer individualized HEPs or harness design features available in mHealth systems to encourage self-management and adherence [11,39]. Going forward, app developers should focus on the inclusion of features that can be specific and customized to the end user for the capabilities of mHealth to be capitalized upon in rehabilitative medicine.

This study reveals a lack of evidence supporting the use of these apps, with only 11.1% (n=16) providing supporting research in their marketing material. Perhaps most concerning is the 14.6% (n=21) of apps that make claims relating to being evidence based but fail to cite any research; the absence of accessible evidence in any of the marketing material makes it difficult to appraise each offering. This might explain why most physiotherapists report only using apps for administrative purposes and not routinely recommending them to support patients’ HEPs in MSK rehabilitation [40]. Health care professionals must feel confident in the evidence base supporting the app to enhance their clinical judgement in their app selection and encourage adoption [41]. The National Institute for Health and Care Excellence (NICE), in collaboration with the National Health Service (NHS) in the United Kingdom, recently published an Evidence Standards Framework for digital and care technologies [42]. This framework contains a comprehensive list of evidence criteria required for such technologies to be adopted into the UK health system, including both minimum and best practice standards. Such frameworks create an awareness among developers, clinicians, and end users of the various types of evidence required for the effective development and implementation of technology in health care.

Communication is a cornerstone of the patient-physiotherapist relationship; a discrepancy in this alliance is a decisive indicator of nonadherence to HEPs, with poor physician communication increasing the risk of nonadherence by up to 19% [43]. Digital health technologies have a variety of communication methods to employ, from telehealth consultations (offered by only 6 of the included apps) to real-time messaging platforms, such as SMS text messaging, emails, or instant messaging (15 apps). The incorporation of such features may encourage the uptake of mHealth apps by clinicians and deviate patients from the more generic “back pain” or “shoulder pain” apps that provide automated programs in the absence of clinician input. The findings from this study are consistent with other research, as physiotherapists expressed concerns about app quality, patient safety, and knowledge base of mHealth apps [38]. A good HEP considers the individual it aims to help, which is fundamental to positively impacting adherence [44]. The literature makes a clear stance in favor of frequent and clear 2-way communication between the therapist and the patient [45], yet less than a quarter of the apps included in this study facilitated communication between the therapist and the patient. 

Facilitating 2-way feedback (patient to clinician and clinician to patient), although challenging, is key to ensure that the clinician is readily equipped with data that can improve clinical decision making [11]. Consumer adoption of digital technology presents an opportunity to continuously capture feedback from patients through clinically approved PROs [46]. A variety of PROs, including standardized instruments, have been developed and validated to use as part of patient management [47], and such features improve communication and enhance clinical decision making [48]. Standardized PROs were featured in less than 15% (n=21) of the apps in this study, something that potentially contradicts the purpose of these “patient-centered” apps. Equally, the delivery of feedback to patients provides an opportunity for the therapist to reassure and educate the patient. The information a patient receives and the beliefs they hold about their condition influence their decision making and thus their adherence [49]. App developers may potentially be adopting the rationale that the inclusion of communication and feedback features may raise concerns regarding patient data security and privacy, with unencrypted communication and third-party data hosting common in general apps in the Google Play Store [50]. Numerous studies have identified the increasing amount of sensitive data handled by mHealth apps as new developments in the industry emerge [51], and this poses challenges to developers and regulators alike.

The idea of using an app in exercise rehabilitation is not to replace the therapist but rather to be seen as a facilitator [52]. mHealth apps have the capacity to send adherence reminders and notifications directly to the device, but the results of this study indicate that this is an area that developers are slow to take advantage of, with just over one-third of the apps featuring adherence reminders. Technology has the potential to affect the outcomes of HEPs by improving the accuracy, adherence, and quality of exercises performed by the patient through multimedia versions of a program (pictures and videos). The inclusion of pictures in a HEP is common in clinical practice [53], although providing patients with videos is slightly more difficult without the use of an app. Remarkably, one-third of the apps failed to incorporate videos into their HEPs [54]. The significant absence of these features, which have shown to increase levels of patient adherence [55], is an area we identified as an underutilization of the resources offered by mHealth apps. 

Health care apps have become an industry in themselves for developers, investors, and health care professionals alike [56]. The findings in this study suggest that for these apps to be used in routine MSK practice, greater efforts need to be made by app developers to engage with academic research and stakeholders. Both health care providers and organizations have quality and validity concerns when it comes to choosing an app to recommend [57]. The absence of features proven to enhance adherence to HEPs, along with no real-time clinician input, leads to the information provided on these apps remaining static [27]. The findings in this study are consistent with those of apps to improve a patient's adherence to medications, with the majority lacking desirable features and considered to be of low quality [58]. There is a wide selection of tools to assess the quality of health-related websites; however, the same cannot be said when it comes to assessing and evaluating mHealth apps [57]. The Mobile App Rating Scale (MARS) is a tool for classifying and assessing the quality of mobile health apps. Further work should look at developing similar tools with a specific relevance to certain areas of health care such as rehabilitation [59]. It would be beneficial for future work to offer stakeholders an informative repository evaluating mHealth apps.

It was beyond the scope of this study to obtain access to the cohort of apps requiring payment to download or private subscriptions. In such cases, data extraction was completed via the app store through analysis of the available screenshots and developer websites. Where evidence of a feature could not be found using this method, it was stated that the feature was absent for this app. As highlighted by Giunti et al [27], while it is possible that an app’s features may only be disclosed to registered app users, this seems unlikely to be a common occurrence as the app store’s description and screenshots serve as major selling points to potential users.

Only apps available in the Republic of Ireland were included in this study. Hence, it is possible that there exists a cohort of eligible apps that have not been included due to geographical limitations. We also decided to exclude white labeled apps. Apps were considered to be white labeled if they were identified as identically structured apps provided by a single developer to multiple different companies. Given that the only discrepancy identified was in accessibility (customers must be linked to the specific private practice or company selling the app to gain access), we felt that the inclusion of such apps would provide a less relevant data set with heavily skewed results. These limitations aside, the data set reported upon reflects the most accurate depiction of the currently available apps for MSK rehabilitation across the 2 major app stores.

It is not surprising that as the capabilities of technology in health care grow, the number of apps coming onto the mHealth app market correlates. Just under 85% (n=122) of the apps that met the inclusion criteria were released into the respective app stores from 2015. The change in outpatient service delivery from traditional face-to-face patient contact to remote management has accelerated rapidly in response to the COVID-19 pandemic [60]. This shift toward technology was reflected in our findings, with 18% (26 apps) of the apps coming to the market in 2020. The pandemic has provided an opportunity for clinicians to embrace innovation and redesign their services to enhance their efficacy beyond the immediate crisis [61-63]. With the rapid proliferation of apps being brought to the market, the findings of this review highlight an opportunity is not being embraced to its full extent. Further research is required to investigate which digital health care features have a meaningful effect on adherence to HEPs. A framework to guide clinician and patient selection of mHealth apps in MSK rehabilitation could help navigate through the overwhelming number of apps available in the respective stores.

### Conclusions

This study analyzed a large number of MSK rehabilitation apps available to consumers. Most of the apps were designed to provide HEPs and empower patients with the aim of improving adherence to HEPs and bridging the gap between the clinic and home. With the emerging capabilities and developments of mHealth, the use of apps in clinical practice is becoming more widely accepted. However, this study identified several missed opportunities by app developers to offer key features that promote adherence and self-management. There was a significant absence of properly cited sourced material or references in the apps included in this study. With the capabilities of mHealth underutilized in physical medicine, this review raises questions about the efficacy and quality of MSK rehabilitation apps, indicating that the current ecosystem of mHealth apps available do not lend well to evidence-based clinical practice. The paucity of evidence in this field reiterates the need for high-quality research and presents an opportunity to all stakeholders involved to develop and enhance these patient-facing apps to further bridge the gap between the clinic and the home.

## Appendix

Multimedia Appendix 1 Featured descriptors.

Multimedia Appendix 2 Additional features.

## Abbreviations

HEP: home exercise program
MARS: Mobile App Rating Scale
mHealth: mobile health
MSK: musculoskeletal
NHS: National Health Service
NICE: National Institute for Health and Care Excellence
PRO: patient-reported outcome
WHO: World Health Organization
