# Supplementary material for: mHealth Apps for Musculoskeletal Rehabilitation: Systematic Search in App Stores and Content Analysis
Source: JMIR Rehabil Assist Technol. 2022 Aug 1;9(3):e34355. doi: 10.2196/34355 (PMC9379789; doi:10.2196/34355)
Supplement: Multimedia Appendix 1 [file rehab_v9i3e34355_app1.docx]

## MULTIMEDIA APPENDIX 1: Feature Descriptors

| **Data Extraction** |
| --- |
| 1. **Year of Release:** Name of the app as it appears on the store. |
|  |
| 1. **Developer:** Developer name as it appears on the store. |
|  |
|  |
| 1. **Charging Model:** |
| - 1. Clinic charged - free for the patient: Clinic pays for use of the software and provides the patient with an access code/password to use the app. |
| - 1. Download charge for the patient: Patient pays to download the app and then has full access to the app. |
| - 1. Free to download with an in-app purchase: Free to download but requires a payment to access certain features of the app. |
| - 1. Free to download and no in app purchase: Completely free access to the app. |
| - 1. Unable to determine. |
| - 1. Multiple options available: Some apps may be free to download and give access to a limited number of features and then require payment for full access or ‘premium’ subscription. Other apps allow one-week free trial and then require payment for access to the apps. |
|  |
| 1. **Exercise Prescription**: |
| - 1. Prescribed by physiotherapist post assessment: Patient has an initial consultation with a therapist and based on this, the therapist prescribes an individualised exercise programme to the patient. |
| - 1. Generic: The patient enters symptoms/location of injury and based on this information a generic exercise programme or bank of exercises is provided to the user. |
| - 1. Both: Includes an option for generic exercise prescription and an individualised programme by a physiotherapist post-assessment. |
|  |
| 1. **Exercise Videos:** Yes/No- Are there videos of the exercises available on the app. |
|  |
| 1. **Exercise Pictures:** Yes/No- Are there pictures of the exercises available on the app. |
|  |
|  |
| 1. **Self-Reported Exercise Log:** Yes/No- Is there an option for the patient to manually report when their exercises are completed. |
|  |
| 1. **Patient Reported Outcomes:** |
| - 1. Respond to targeted questions: The app asks the patient how they are feeling, what their mood/pain is like etc. |
| - 1. Standardised Instruments: Patient fills out questionnaires, visual analogue scales etc. |
| - 1. Free text: Patient can report on any aspect of their performance if they choose. |
| - 1. Present but unable to tell: From the data extraction it is evident that there are patient reported outcomes but cannot distinguish which form it takes. |
| - 1. Multiple Options: Includes two or more of the options above. |
| - 1. None. |
|  |
| 1. **Communication:** |
| - 1. Two-way messaging: The patient can send a message to their physiotherapist via the app and the physiotherapist can respond at a time convenient to them. |
| - 1. Instant messaging: live chat feature in app where patient and physio can communicate if both are online at the same time. |
| - 1. Robotic: Automated response to questions or use of a virtual trainer. |
| - 1. Video conferencing: The physio and patient can have an online video consultation through the app. |
| - 1. Unable to determine: Communication feature present but unable to differentiate which method is being used. |
| - 1. Combination: App incorporates 2 or more of the communication features mentioned above. |
| - 1. None. |
|  |
| 1. **Feedback to the Patient:** |
| - 1. Automated: sends automatic feedback regarding sessions remaining, amount completed etc. |
| - 1. Direct feedback from the physiotherapist: The physio gives individualised feedback directly to the patient via the app. |
| - 1. Gamification: The app gives the patient rewards or badges for achieving goals or completing exercises. |
| - 1. Progress Tracking: The app produces statistics or graphs based on the patient’s inputs. |
| - 1. Multiple: The app contains two or more of the feedback features outlined above. |
| - 1. None. |
|  |
| 1. **Adherence Reminders:** Yes/No- Does the app send push notifications to the smartphone to remind patients to complete their exercise programme. |
|  |
| 1. **Research Paper References/Evidence:** |
| - 1. Reference provided to research: References to research pertaining to the app can be found in the app description or on the app website. |
| - 1. Scientific evidence claims: Claims made by the developers that are not backed up by any evidence or references. |
| - 1. No Research referred to: No evidence of research in the app description or the app website. |
|  |
| 1. **Targeted Body Part:** |
| - 1. Tailored: Exercises tailored to an individual body part after physiotherapist assessment or following inputs of a specific injury into the app. |
| - 1. Specific Body Part: Knee, ankle, hand, hip etc. |
|  |
| 1. **Patient Education:** Yes/No- Does the app have information/tips for the patient about managing their condition/injury. |
|  |
| 1. **Safety Features:** Yes/No- Does the app have disclaimers to consult with a healthcare professional or list any contraindications or precautions for the patient. |
