# Supplementary material for: mHealth Apps for Musculoskeletal Rehabilitation: Systematic Search in App Stores and Content Analysis
Source: JMIR Rehabil Assist Technol. 2022 Aug 1;9(3):e34355. doi: 10.2196/34355 (PMC9379789; doi:10.2196/34355)
Supplement: Multimedia Appendix 2 [file rehab_v9i3e34355_app2.docx]

## MULTIMEDIA APPENDIX 2

**S1.** Prevalence of Exercise-Based Features Within Applications (n=144)

| **Feature** | **Number of Applications, n (%)** |
| --- | --- |
| **Exercise Videos** |  |
| Yes | 97 (67.4) |
| No | 17 (32.6) |
| **Exercise Pictures** |  |
| Yes | 138 (95.8) |
| No | 6 (4.2) |
| **Self-Reported Exercise Log** |  |
| Yes | 66 (45.8) |
| No | 78 (54.2) |
| **Mode of Exercise Prescription** |  |
| Automated Prescription | 93 (64.6) |
| Prescription Post Physiotherapy Assessment | 44 (30.6) |
| Both Modes Present | 7 (4.9) |
| **Targeted Body Part** |  |
| Tailored | 78 (54.2) |
| Ankle | 2 (1.4) |
| Back | 18 (12.5) |
| Back & Core | 1 (0.7) |
| Back & Hip | 1 (0.7) |
| Back & Knee | 2 (1.4) |
| Back & Neck | 2 (1.4) |
| Hand | 3 (2.1) |
| Hand & Wrist | 1 (0.7) |
| Knee | 21 (14.6) |
| Knee & Hip | 2 (1.4) |
| Neck | 5 (3.5) |
| Neck & Shoulder | 2 (1.4) |
| Shoulder | 6 (4.2) |

**S2.** Additional Features Identified in Applications (n=144)

| **Feature** | **Number of Applications, n (%)** |
| --- | --- |
| **Communication** |  |
| Video Conferencing | 6 (4.2) |
| Messaging |  |
| Two-Way Messaging | 8 (5.6) |
| Instant Messaging | 4 (2.8) |
| Robotic Messaging | 1 (0.7) |
| Messaging, Unable to Discern Type | 2 (1.4) |
| None | 112 (77.8) |
| Multiple Sources | 11 (7.7) |
| **Patient-Reported Outcomes** |  |
| Free Text | 1 (0.7) |
| Targeted Questioning | 2 (1.4) |
| Standardised Instruments | 21 (14.6) |
| Multiple Sources | 11 (7.6) |
| None | 108 (75) |
| Present, Unable to Discern Type | 1 (0.7) |
| **Feedback to Patient** |  |
| Direct Feedback from Physiotherapist | 5 (3.5) |
| Progress Tracking | 25 (17.4) |
| Automated Feedback | 5 (3.5) |
| Gamification | 5 (3.5) |
| Multiple Sources | 17 (11.8) |
| None | 87 (60.4) |
| **Patient Education** |  |
| Yes | 49 (34) |
| No | 95 (66) |
| **Adherence Reminders** |  |
| Yes | 49 (34) |
| No | 95 (66) |
| **Safety Advice & Disclaimers** |  |
| Yes | 38 (26.4) |
| No | 106 (73.6) |
